# Applying Implementation Mapping to Expand a Care Coordination Program at a Federally Qualified Health Center

**DOI:** 10.3389/fpubh.2022.844898

**Published:** 2022-03-24

**Authors:** Kelsey S. Dickson, Tana Holt, Elva Arredondo

**Affiliations:** ^1^Department of Child and Family Development, San Diego State University, San Diego, CA, United States; ^2^Child and Adolescent Services Research Center, San Diego, CA, United States; ^3^San Diego State University Research Foundation, San Diego State University, San Diego, CA, United States; ^4^Department of Psychology, San Diego State University, San Diego, CA, United States; ^5^Institute for Behavioral and Community Health, San Diego State University, San Diego, CA, United States

**Keywords:** Implementation Mapping, care coordination, federally qualified health center, evidence-based practice, implementation strategy

## Abstract

**Background:**

A large and growing percentage of medically underserved groups receive care at federally qualified health centers (FQHCs). Care coordination is an evidence-based approach to address disparities in healthcare services. A partnered FQHC established a care coordination model to improve receipt and quality of healthcare for patients most at risk for poor health outcomes. This care coordination model emphasizes identification and support of behavioral health needs (e.g., depression, anxiety) and two evidence-based behavioral health programs needs were selected for implementation within the context of this care coordination model. Implementation Mapping is a systematic process for specifying the implementation strategies and outcomes. The current case study describes the application of Implementation Mapping to inform the selection and testing of implementation strategies to improve implementation of two behavioral health programs in a Care Coordination Program at a partnered FQHC.

**Methods:**

We applied Implementation Mapping to inform the development, selection and testing of implementation strategies to improve the implementation of two evidence-based behavioral health programs within a care coordination program at a partnered FQHC.

**Results:**

Results are presented by Implementation Mapping task, from Task 1 through Task 5. We also describe the integration of additional implementation frameworks (The Consolidated Framework for Implementation Research, Health Equity Implementation Framework) within the Implementation Mapping process to inform determinant identification, performance and change objectives development, design and tailoring of implementation strategies and protocols, and resulting evaluation of implementation outcomes.

**Conclusions:**

The current project is an example of real-world application of Implementation Mapping methodology to improve care outcomes for a high priority population that is generalizable to other settings utilizing similar care models and health equity endeavors. Such case studies are critical to advance our understanding and application of innovative implementation science methods such as Implementation Mapping.

## Introduction

Profound disparities in accessing and receiving quality healthcare exist for Hispanic or Latino/a individuals, likely contributing to the unequal rates of health issues spanning multiple health areas (e.g., health status, acute and chronic diseases, behavioral health) (1–4). Among these are higher rates of behavioral health conditions and unmet mental health needs when compared to White individuals, conferring vulnerability to further medical and behavioral health problems, preventable morbidity, and societal cost (1, 5). These care disparities have immense public health implications given that the Hispanic or Latino/a population represents the largest and most rapidly growing minority population in California and the United States (6). Efforts to promote equitable and effective care are critical to improve the health of this increasing population and diminish the associated public health impact. Given both the prevalence of behavioral health conditions and substantial public health impact, behavioral health represents a key target within healthcare and health equity efforts.

Federally qualified health centers (FQHCs) play a significant role in the care provision of largely underserved populations, especially Hispanic or Latino/a individuals. FQHCs are funded to provide health care, including primary care and related services, in underserved areas to offset multiple barriers (e.g., geographic, cultural) in care access and utilization. Data suggest that traditionally marginalized individuals, including lower income, racial and ethnic minority or uninsured individuals, comprise a large and increasing portion of those served by FQHCs (7). Further, Hispanic or Latino/a individuals comprise as much as 38% of those served by FQHCs (8, 9), making FQHCs uniquely positioned to promote health and healthcare equity for this population. Importantly, the prevalence of behavioral health conditions among patients are higher in FQHCs compared to other settings (10), with data suggesting that behavioral health conditions such as depression or anxiety were the third most frequent condition seen in FQHCs in 2020 (11). These higher rates of behavioral conditions further underscore the importance of ensuring FQHCs are equipped to address the behavioral health needs of patients served as part of the broader care provision model.

Care coordination is an evidence-based care model that is increasingly implemented to improve care equity, including in FQHCs (12–14). Defined as a person-centered, interdisciplinary approach to integrating healthcare, care coordination models involve case managers to integrate and support patient care, including services from primary care and other care specialists, patient education and treatment management, adjustment, and follow-up (12–14). Care Coordinators identify the specific needs of patients and the services they are receiving to ensure communication across the multiple service providers and to provide patient education and support surrounding treatment goals and recommendations (15–19). Such models can help bridge key care gaps to improve health equity and are increasingly recommended given their effectiveness for patients with co-occurring medical and behavioral health conditions (19, 20). Indeed, a focus on behavioral health needs is a key qualification area for care coordination accreditation models (21). Further, data support the effectiveness of collaborative care models in treating depression among low-income and minority communities, including Hispanic or Latino/a individuals (19, 22).

In 2017, a partnered FQHC implemented a care coordination model to support health promotion among most at-risk patients. Given the location along the US-Mexico border, most patients served are Hispanic or Latino/a, living at or below 200% of the federal poverty line, and/or largely uninsured. Consistent with broader accreditation standards, behavioral health conditions are a qualifying condition for the care coordination program as well as a prioritized health target of the broader organization. Training in evidence-based behavioral health programs is provided as part of this program, including training in two well recognized and federally and locally prioritized evidence-based practices (EBPs), Mental Health First Aid (23–25) and the Adverse Childhood Experiences Screener (26). Mental Health First Aid is an educational program to increase mental health literacy, reduce stigma, and support mental health service navigation. Through didactic training, implementers are provided with a broad knowledge of behavioral health conditions and basic skills in recognizing, approaching and providing initial support for behavioral health problems (23). The Adverse Childhood Experiences Screener is a short questionnaire used to rapidly identify and assess patients that may be at risk for poor health outcomes due to childhood trauma (26). To optimize implementation and effectiveness of these programs and improve both implementation and patient health outcomes, we applied Implementation Mapping to support an effort to expand and support implementation of behavioral health EBPs within the context of this Care Coordination program serving patients with chronic health condition (e.g., Diabetes, hypertension) at the partnered FQHC.

### Implementation Mapping

Informed by the Intervention Mapping process and implementation science, Implementation Mapping provides step-by-step guidance for selecting and designing implementation strategies to guide implementation efforts (27). Implementation Mapping details five sequential tasks: (1) conduct a needs assessment; (2) identify implementation outcomes and performance objectives, identify determinants, and create matrices of change objectives; (3) identify and select theoretical methods implementation strategies; (4) create implementation protocols and materials; and (5) evaluate implementation. Consistent with the Intervention Mapping process on which it was based, Implementation Mapping facilitates implementation strategy development and selection that appropriately consider and address contextual needs and determinants, thereby optimizing implementation outcomes (27). In the current case study, Implementation Mapping in conjunction with broader implementation frameworks, including those specifying key health equity domains, will allow for identification of organizational and provider specific strategies to support EBP implementation and consider key implementation and care equity barriers (e.g., stigma, limited awareness) common to implementing behavioral health programs in settings like the partnered FQHC (28–30).

The purpose of this manuscript is to present a case study featuring the application of Implementation Mapping as part of a study that aims to examine the implementation and expansion of an existing, community-initiated health equity effort within a FQHC located along the US-Mexico border. In combination with relevant health equity and determinant implementation frameworks, we utilized the Implementation Mapping process to inform the development, selection and testing of different strategies to expand and enhance the implementation of evidence-based behavioral health programs within the Care Coordination program at a partnered FQHC.

## Methods

This study is supported as part of the NIMHD-funded San Diego State HealthLINK Center for Transdisciplinary Health Disparities Research (U54MD012397; PIs: Ayala, Wells) aiming to enhance community capacity and improve infrastructure to advance minority health and health disparities. This project focuses on adapting and developing behavioral health evidence-based practice components and corresponding implementation strategies to expand and facilitate delivery of existing evidence-based behavioral health programs implemented within an existing care coordination model at a FQHC. This study was conducted in collaboration with key stakeholders at the FQHC, particularly those involved with the Care Coordination program, and investigators who have extensive experience working with Hispanic or Latino/a communities (E.A.). These individuals provided input and guidance for the design and selection of implementation strategies. This study was approved from the Institutional Review Board (IRB) at the academic institution as well as the *ad-hoc* IRB at partnered FQHC. Informed consent was obtained from all participants in the current project.

### Guiding Implementation Frameworks

In addition to the Implementation Mapping Process, we applied the Consolidated Framework for Implementation Research [CFIR; (31)] to guide the current study. We selected CFIR given the interest in examining organizational level determinants, specification of key implementation determinants, and utility in prior programs conducted in FQHCs applying the Implementation Mapping process [e.g., (32)]. Given the specific emphasis on health equity in the current project, we also applied the Health Equity Implementation Framework [HEIF; (33, 34)] to enable examination of key implementation determinants that may explain the social determinants of health. Specifically, we integrated the three health equity domains detailed within this framework into our application of CFIR.

## Results for the Application of Implementation Mapping

### Implementation Mapping Task 1: Conduct a Needs Assessment

The first aim of this study consisted of a sequential mixed-methods (quan-QUAL) needs assessment to identify care coordinator perspectives regarding: (1) client service and Care Coordinator training needs related to behavioral health; (2) implementation determinants for selected evidence-based behavioral health programs; and (3) necessary modifications or enhancements to selected evidence-based behavioral health programs. We also assessed perceptions regarding existing and potentially relevant implementation strategies via our initial quantitative survey. The selected implementation frameworks (CFIR, HEIF) guided data collection, analyses, and interpretation, including application to iteratively develop and refine a qualitative focus group guide and codebook applied to conduct and analyze focus groups through in-depth coding. Consistent with the HEIF, for example, we included an explicit emphasis on culturally relevant factors and determinants through specific focus group questions, probes, and codebook. We also included questions pertaining to the CFIR constructs of behavioral health knowledge and beliefs and compatibility of existing evidence-based behavioral health programs such as “Given your experience with these programs, how well do these programs fit with or are appropriate for [the needs of your patients, your role as a care coordinator, the realities of your organization]?” We then included an additional probe assessing for the HEIF health equity domain of cultural relevance, including the fit or acceptability of these practices with the culture, beliefs, preferences and/or language of the largely Hispanic or Latino/a patients served.

Participants included Care Coordinators (*n* = 8 or 50% of the broader population of Care Coordinators at the FQHC) who participated in the initial web-based survey and subsequent virtual focus group; the pilot project lead (K.D.) with experience in mixed-methods needs assessment and qualitative methods led the focus groups. Each focus group lasted approximately 45 min and were conducted in English *via* secure videoconferencing software (i.e., HIPAA-compliant Zoom). The majority of participants were female (75%), with a Bachelor's (63%) or Associate's (38%) Degree. All identified as Mexican or of Mexican descent and reported delivering care coordination services in English and Spanish. The pilot project lead (K.D.) also conducted two informational interviews with FQHC leaders to gather necessary information regarding evidence-based practice decision making and identification of relevant processes and resources. Qualitative data were initially analyzed using rapid assessment process (35, 36), with findings categorized following each focus group in alignment with focus group guide domains specified by CFIR and HEIF. We (K.D. and T.H.) conducted subsequent in-depth consensus coding, applying an iteratively developed codebook informed by a priori and emergent themes and the guiding frameworks. The codebook contained definitions of the codes and guidelines for use. We integrated both quantitative and qualitative types to examine complementarity and expansion (37).

Results from our needs assessment indicated multilevel determinants spanning the organizational, implementer and end recipient or patient levels, including perceived client service and Care Coordinator training needs, for consideration. This suggested a need for multilevel performance objectives to best address these needs and achieve outcomes (see Task 2). Findings indicated limited behavioral health knowledge among both patients and Care Coordinators as well as Care Coordinator limited self-efficacy addressing or assessing behavioral health concerns and implementing behavioral health EBPs. Importantly and consistent with HEIF, our results also indicated several culturally relevant factors or determinants that were raised several times throughout both focus groups. This included the cultural stigma commonly associated with behavioral health and behavioral health treatments within the Mexican culture. A poor match between care practices or recommendations and cultural values was also described. For example, several participants described preferences or beliefs regarding alternative or traditional treatments among their patients frequently limit or impeded adherence to additional treatment recommendations. At the organizational level, limited collaboration between Care Coordinators and behavioral health providers as well as challenges related to the availability of behavioral health services emerged as barriers to EBP implementation. Results also indicated several relevant strategies to address these determinants, including ongoing, dynamic behavioral health trainings, additional culturally relevant and tailored behavioral health educational materials for both patients and Care Coordinators and increased collaboration between Care Coordination and behavioral health. Following analyses, we shared our results with our FQHC partners to aid further contextualization and interpretation and used them to inform identification of relevant outcomes, performance objectives and change objectives (Task 2) as well as selection and design of implementation strategies (Task 3).

In collaboration with our FQHC partners, our needs assessment also informed and confirmed those involved in the implementation of the evidence-based program and those required to support execution of the corresponding implementation plan. We confirmed that Care Coordinators would be the primary program implementers given the alignment between the evidence-based program target of behavioral health and workload responsibilities and expectations surrounding behavioral health for Care Coordinators. Care Coordination and organizational leaders would facilitate execution of the implementation strategies identified in Task 2. While the initial evidence-based behavioral health trainings would be facilitated by the research team, trainings were designed to be sustainable such that Care Coordination leaders can continue to facilitate and conduct these trainings following the completion of the study.

### Implementation Mapping Task 2: Identify and State Adoption and Implementation Outcomes, Performance Objectives, Determinants, and Change Objectives

As mentioned, Task 1 findings aided the identification of relevant implementation outcomes, performance objectives corresponding to each identified implementation outcome, determinants of each performance objective, and change objectives mapped onto identified performance objectives and determinants. In collaboration with FQHC partners, we identified relevant implementation outcomes as well as necessary performance objectives to achieve these outcomes. The project lead and coordinator then reviewed the preliminary needs assessment findings to identify multilevel determinants relative to these performance objectives. Importantly, our Task 1 needs assessments identified several determinants, especially those pertaining to broader outer context or community-level, that while relevant, were deemed not directly relevant to our stated performance objectives and outside the scope of the current project. Thus, these were not included among our final determinants. This included barriers not directly related to behavioral health needs such as social service offerings (e.g., food distributions) or cultural food preferences that were incompatible with broader medical care or medically-related Care Coordination goals (e.g., limiting high carb such as those common in non-perishable foods).

Determinants were also informed by broader CFIR and HEIF health equity domains to ensure alignment with our guiding implementation theories. For example, our needs assessment findings suggested limited knowledge and efficacy surrounding behavioral health. Consistent with the CFIR inner context domains Knowledge and Beliefs and Personal Attributes, this contributed to our specification of behavioral health knowledge and efficacy determinants. Additionally, and consistent with the HEIF health equity domain of culturally relevant factors, we identified knowledge and self-efficacy related to culturally relevant resources and practices as important determinants of stated performance objectives. Finally, we identified change objectives tied to each performance objective and determinant selected. See Table 1 for summary of implementation outcomes, performance objectives and relevant determinants.

**Table 1:** Implementation outcomes with corresponding performance and determinants.

**Implementation outcomes**		**Determinants (mapped onto CFIR and HEIF domains in parentheses)**		
	**Performance objectives**	**Knowledge (CFIR-knowledge and beliefs; HEIF-cultural relevance)**	**Skills and self-efficacy (CFIR-personal attributes)**	**Outcome expectations (CFIR-compatibility; personal attributes; relative priority)**
**Care coordinators**				
Implementation: • Care coordinators implement behavioral health EBP strategies • Care coordinators will follow identified EBP implementation workflows and procedures (e.g., screen for behavioral health) Sustainability: • Care coordinators continue using behavioral health EBPs with patients	PO.1: Utilize behavioral health EBP strategies, including culturally relevant strategies, to support recognition of signs or symptoms of behavioral health concerns PO.2: Utilize behavioral health EBP strategies to initiate discussion of behavioral health concerns and refer to behavioral health services (if applicable) PO.3: Follow identified EBP workflow and procedures	K.1: Awareness of behavioral health EBP strategies K.2: Awareness of culturally relevant behavioral health resources and practices K.3: Knowledge of caregiver-directed strategies K.4: Awareness of organizational EBP implementation procedures and workflows	SSE.1: Demonstrate ability to deliver and maintain use of behavioral health EBP strategies to address patient behavioral health needs SSE.2: Express confidence in ability to identify and use culturally relevant behavioral health strategies SSE.3: Express confidence using caregiver-directed strategies to increase care engagement SSE.4: Demonstrate ability to navigate and adhere to EBP workflow procedures	OE.1: Expect that EBP training, delivery, and maintenance will better meet patient behavioral health needs and improve care effectiveness OE.2: Expect that culturally relevant resources and practices will improve match between patient cultural values and care OE.3: Expect that caregiver-directed strategy use will improve patient engagement OE.4: Expect that workflows and procedures will aid EBP implementation
**Organization and leaders**				
Adoption: • Provide behavioral health EBP materials Feasibility: • Identify, adapt, and execute necessary EBP implementation procedures and workflows Implementation: • Facilitate ongoing behavioral health EBP trainings and resources Sustainability: • Maintain EBP implementation and workflow procedures	PO.1: Communicate with staff about practice change PO.2: Facilitate EBP materials and ongoing trainings PO.3: Assure procedures in place for EBP implementation PO.4: Assure sustained EBP implementation and corresponding workflow procedures	K.1: Describe process for communicating practice changes K.2: Describe processes for ongoing EBP training K.3: Describe process for ensuring EBP implementation procedures K.4: Describe steps to assure sustained EBP implementation workflow and procedures	SSE.1: Demonstrate administrative ability to communicate planned practice changes SSE.2: Demonstrate administrative ability to facilitate ongoing program EBP trainings SSE.3: Demonstrate administrative ability to maintain EBP implementation procedures SSE.4: Demonstrate administrative ability to maintain ongoing program EBP implementation	OE.1: Expect that practice change communication will improve care coordinator readiness OE.2: Expect that EBP training will improve implementation OE.3: Expect that workflow procedures will improve staff engagement and completion of EBP trainings OE.4: Expect that sustained workflow procedures will improve sustained EBP implementation

### Implementation Mapping Task 3: Change Method and Implementation Strategy Selection and Design

To complete this task, we first developed and selected theoretical change methods expected to target the determinants and change objectives identified in Task 2. This informed the subsequent, iterative selection of implementation strategies that appropriately operationalized our change methods. As in prior Tasks, this process was done in collaboration with our FQHC partners. We began by considering the implementation determinants and change objectives identified in Task 2 and referred to specific Task 1 quantitative results regarding Care Coordinators perspectives of relevant implementation strategies. This led to the development of specific theoretical change methods, informed by our guiding CFIR and HEIF implementation frameworks as well as literature regarding causal theories in implementation science [e.g., (38)]. For example, given the identified role of knowledge and knowledge change in promoting successful adoption and implementation, this was hypothesized as a key change method. To operationalize these change methods, we then developed and selected a list of possible implementation strategies. Informed by CFIR, we then prioritized those methods and strategies that would address implementation determinants toward achieving outcomes across multiple inner context levels, including providing information via training and educational materials targeting behavioral health knowledge and efficacy. We iteratively refined our implementation strategies following feedback from our community partner, including feedback regarding fit and feasibility within their organization (Table 2).

**Table 2:** Sample change objectives with corresponding implementation determinants, methods and implementation strategies.

**Change objective**	**Determinant**	**Theoretical change methods**	**Implementation strategies/practical application**
**Care Coordinators**			
SSE.1: Demonstrate ability to deliver and maintain use of behavioral health EBP strategies to address patient behavioral health needs	• Skills/self-efficacy • Outcome Expectation	• Provide Information • Skill-building and Guided Practice	• Conduct brief face-to-face training incorporated into existing monthly Care Coordinator meetings
K.2: Awareness of culturally relevant behavioral health resources and practices	• Knowledge and Awareness	• Improved knowledge • Provide Information	• Develop and distribute additional culturally relevant, tailored behavioral health materials
**Organization and leaders**			
SSE.2: Demonstrate administrative ability to facilitate ongoing program EBP trainings	• Skills and Self-Efficacy	• Organizational Planning • Technical assistance/Capacity building	• Brief face-to-face behavioral health trainings incorporated into existing monthly Care Coordination meetings
K.4: Describe steps to assure sustained EBP implementation workflow and procedures	• Knowledge and Awareness	• Communication • Organizational Planning	• Meetings to discuss maintaining trainings and EBP implementation workflow maintenance • Facilitate discussion regarding linkage and collaboration with behavioral health

During our design, selection, and refinement of implementation strategies, we were mindful of the specific implementation context and parameters within the partnered FQHC. For example, we considered but ultimately did not include the specific strategies of identifying implementation champions and/or quality monitoring to operationalize our change methods of Skill-building, Guided Practice, and Capacity Building but did not select these as they did not optimally fit with the specific structure and roles of the care coordination program, including Care Coordinator workload expectations and responsibilities. Additionally, we developed and tailored strategies to ensure complementarity with existing strategies utilized. For instance, the partnered FQHC conducted trainings for the selected behavioral health EBP materials with Care Coordinators as well as distributed behavioral health educational materials. To complement these strategies, we designed additional behavioral health educational materials targeting improved behavioral health knowledge and efficacy. Given the health equity focus within this project and consistent with the HEIF, strategies were designed or tailored to address or include culturally relevant factors such as patient beliefs, preferences, and treatment or care expectations. For example, educational materials developed aimed to destigmatize behavioral health and detail what the patient could expect from behavioral health services. To expand on existing EBP trainings, we designed ongoing, dynamic and adaptable trainings that were tailored to the specific needs (e.g., health care needs, cultural) of patients served. Trainings will be supplemented with ongoing implementation support and consultation as needed. Table 3 details the specific implementation strategies selected.

**Table 3:** Final implementation protocol.

**Implementation stage**	**Determinants/change methods**	**Theoretical change methods**	**Implementation strategies** **(E = Existing; A = Added)**	**Practical application**
Adoption	• Knowledge and Awareness • Skills and Self-Efficacy • Outcome Expectations	• Organizational Consultation/ Planning • Information • Persuasion	• Capture and share local knowledge (E) • Develop academic partnership (A) • Conduct local needs assessment (A) • Identify implementation determinants (A)	• Informational interview with care coordinator and organizational leaders • Complete implementation readiness checklist • Review of existing behavioral health educational materials and EBPs • Review of existing behavior health workflows and procedures • Ongoing meetings to support iterative and collaborative development of additional behavioral health EBP materials, workflows, and implementation supports • Needs assessment findings and training plans shared with care coordinators
Implementation	• Knowledge and Awareness • Skills and Self-Efficacy • Outcome Expectations	• Information • Improved Knowledge • Persuasion • Skill building and Guided Practice • Improved Collaboration • Improved Efficacy	• Develop and distribute educational materials (E/A) • Make training dynamic and promote adaptability (A) • Conduct ongoing educational meetings and training (E/A) • Develop and implement tools and procedures for quality monitoring (E/A) • Promote network weaving (A)	• Development and distribute additional culturally relevant, tailored behavioral health materials • Develop tailored, pragmatic behavioral health EBP strategies and training • Brief face to face behavioral health trainings incorporated into existing monthly Care Coordinator • Establish procedures for increased collaboration between Care Coordinator and behavioral health • Establish behavioral health EBP implementation workflows and procedures
Sustainability	• Knowledge and Awareness • Skills and Self-Efficacy	• Information • Organizational Planning • Communication • Technical Assistance/Capacity Building	• Provide ongoing consultation and technical assistance (A)	• Meetings to discuss maintaining trainings and EBP implementation workflow maintenance • Training annotated to support delivery by care coordination leaders and staff • Research team provide ongoing technical assistance and implementation support and available as needed

### Implementation Mapping Task 4: Implementation Protocol and Materials

We finalized the process of identifying and developing implementation strategies (Task 3) to create an implementation protocol. It details the implementation strategies and practical applications, or those more detailed aspects of the implementation strategies, we designed to create change in the implementation determinants and change objectives identified in Task 2. We expect these implementation determinants and change objectives to drive achievement of the performance objectives and influence the specified implementation outcomes. Development of the protocol, activities and materials occurred in collaboration with our community partners to enhance the contextual fit within the organization as well as improve identified implementation strategies. To optimize feasibility and sustainability for example, we designed our ongoing trainings to be brief and pragmatic to permit incorporation into existing Care Coordinator team meetings (vs. requiring identification of additional training time). Psychoeducational and training topics were selected and/or developed to address patient and Care Coordinator behavioral health needs as well as normalize and destigmatize behavioral health. Sample topics included what to expect from behavior health services for patients, evidence-based stress management, coping strategies and patient engagement strategies, psychoeducation for setting behavioral health-oriented treatment goals, and psychoeducation for addressing and preventing secondary trauma. Further, we annotated all materials to enable ongoing delivery by partnered Care Coordinator leaders and/or staff as needed.

### Implementation Mapping Task 5: Evaluate Implementation Outcomes

Implementation evaluation is planned as part of an ongoing preliminary pilot test of the selected EBP components and implementation protocol within the context of the partnered Care Coordinator program. Evaluation of identified strategies and associated impact on determinants and implementation outcomes is planned using a mixed-methods (quan->QUAL) approach. Initial quantitative measures will assess feasibility, acceptability, and appropriateness, as well as Care Coordinator knowledge and efficacy surrounding behavioral health using existing measures [e.g., Feasibility of Intervention Measure, Acceptability of Intervention Measure and Intervention Appropriateness measure (39); adapted evidence-based practice knowledge and confidence measure (40)] tailored for the current study. Qualitative interviews will expand on quantitative data regarding implementation outcomes as well as explore participating Care Coordinator perspectives' regarding programmatic impact on patient-level determinants and outcomes. Again, data collection and analyses will be guided by CFIR and the HEIR. Similar to our Task 1 needs assessments, questions will assess the compatibility of the developed evidence-based behavioral health practices and strategies as well as implementation strategies, including questions such as “You mentioned in the survey that you found the specific strategy of [insert strategy identified in quantitative survey here] as helpful. Can you tell us how you found this helpful?” with the specific probes regarding the cultural relevance and/or fit of this strategy with patients. We anticipate analyzing data using similar methods as in our Task 1 mixed-methods needs assessment.

## Discussion

Implementation Mapping has the potential to respond to the need for enhanced methods to design, tailor, test, and evaluate implementation strategies in service of improving effective care delivery and outcomes in community settings (41). Indeed, prior work as well as the work included within this special issue highlight its utility in applying this approach to develop and test implementation strategies to improve the translation of effective care practices (27, 32). The current work presented a case study of ongoing work to apply Implementation Mapping to inform implementation strategy development to expand an existing community-initiated health equity initiative at a partnered FQHC.

A particular strength of the Implementation Mapping approach is the systematic approach to developing and tailoring implementation strategies and materials that begins with articulating desired outcomes and works in a stepwise, linked fashion toward describing behaviors and behavioral determinants associated with those outcomes. This allowed for facile application of this process as part of community-identified implementation effort where outcomes, especially service outcomes, were already selected and prioritized. In the current project, that included improving the health outcomes, especially behavioral health outcomes, of patients served in the Care Coordination program. An additional strength of this approach is the ease of incorporation of additional implementation science frameworks within the Implementation Mapping process. Given the explicit focus on health equity and organizational implementation determinants in the current study, for example, the application of CFIR and health equity domains from the HEIF was necessary for the current project. Finally, the current project demonstrates the immense utility of applying the Implementation Mapping to advance health equity implementation efforts given the strong emphasis on identifying and addressing implementation determinants, including those contributing to ongoing healthcare inequities, throughout each stepwise task.

This case study also underscored the importance of incorporating strong community partnerships as part of the Implementation Mapping process. The continued input and feedback obtained from our partners and leaders at the FQHC was invaluable to our application of Implementation Mapping, particularly during the selection and design of implementation strategies and methods (Task 3) to assure the feasibility and appropriateness within their organizational context and existing implementation strategies. The value added of involving community stakeholders is consistent with its role as an integral component of implementation and consideration as best practice for implementation research (42, 43). Community engagement adds additional value as part of implementation science methodologies such as Implementation Mapping through by assuring that the continued development and application of these methodologies align with community originated implementation initiatives such as the care coordination program of interest in the current study.

We noted some limitations to Implementation Mapping process, namely the time intensive nature of this process. As noted, the application of this process spanned multiple months, which is consistent with similar work noting a similar timeline as well as large number of individuals involved (32, 44). While these limitations certainly do not outweigh the immense benefits resulting from this process, the time and resources necessary may preclude its use in projects that may otherwise greatly benefit but lack these resources, including community-initiated implementation projects. Future directions include additional application of Implementation Mapping, especially within the context of rapid implementation projects or those applying more rapid implementation methods to better understand its use and utility in such projects.

## Conclusions

There is a need for more systematic selection, design, specification, and testing of implementation strategies, including methods and tools to support doing so, to maximize the successful translation of EBPs. Implementation Mapping represents a practical method that has the potential to advance our use and understanding of implementation strategies. The current study provides a case study of the application of Implementation Mapping to an applied, community-partnered project aiming to examine the implementation and expansion of an existing, community-initiated health equity effort within a FQHC. It may provide useful insights for future work aiming to apply the Implementation Mapping process to support further health equity implementation efforts.

## Data Availability Statement

The raw data supporting the conclusions of this article will be made available by the authors, without undue reservation.

## Ethics Statement

The studies involving human participants were reviewed and approved by San Diego State University. The patients/participants provided their written informed consent to participate in this study.

## Author Contributions

KD and TH wrote sections of the manuscript. KD and EA participated in the development of implementation mapping process. All authors reviewed and edited the manuscript.

## Funding

Research reported in this manuscript was supported by the National Institute on Minority Health and Health Disparities, part of the National Institutes of Health, under award number U54MD012397. KD was also supported by a National Institute of Mental Health Career Development Award K23 MH115100.

## Author Disclaimer

The research content is solely the responsibility of the authors and does not necessarily represent the official views of the National Institutes of Health.

## Conflict of Interest

The authors declare that the research was conducted in the absence of any commercial or financial relationships that could be construed as a potential conflict of interest.

## Publisher's Note

All claims expressed in this article are solely those of the authors and do not necessarily represent those of their affiliated organizations, or those of the publisher, the editors and the reviewers. Any product that may be evaluated in this article, or claim that may be made by its manufacturer, is not guaranteed or endorsed by the publisher.
